# Combinatory Nanovesicle with siRNA-Loaded Extracellular Vesicle and IGF-1 for Osteoarthritis Treatments

**DOI:** 10.3390/ijms25105242

**Published:** 2024-05-11

**Authors:** Jun Yong Kim, Seung Yeon Lee, Seung-Gyu Cha, Jung Min Park, Duck Hyun Song, Sang-Hyuk Lee, Dong-Youn Hwang, Byoung Ju Kim, Seungsoo Rho, Chun Gwon Park, Won-Kyu Rhim, Dong Keun Han

**Affiliations:** 1 Department of Biomedical Science, CHA University, 335 Pangyo-ro, Bundang-gu, Seongnam-si 13488, Republic of Korea; jy8050@skku.edu (J.Y.K.); totoro0218@naver.com (S.Y.L.); chkh8047@naver.com (S.-G.C.); 3089pjm@naver.com (J.M.P.); mondh920@naver.com (D.H.S.); humit159@naver.com (S.-H.L.); hdy@cha.ac.kr (D.-Y.H.); 2 Department of Biomedical Engineering, SKKU Institute for Convergence, Sungkyunkwan University (SKKU), 2066 Seobu-ro, Jangan-gu, Suwon-si 16419, Republic of Korea; chunpark@skku.edu; 3 Intelligent Precision of Healthcare Convergence, SKKU Institute for Convergence, Sungkyunkwan University (SKKU), 2066 Seobu-ro, Jangan-gu, Suwon-si 16419, Republic of Korea; 4 ATEMs, Jeongui-ro 8-gil, Songpa-gu, Seoul-si 05836, Republic of Korea; kbz9861@hanmail.net; 5 Department of Ophthalmology, CHA Bundang Medical Center, CHA University, Seongnam-si 13496, Republic of Korea; harryrho@gmail.com

**Keywords:** extracellular vesicles (EVs), mesenchymal stem cell (MSC), siRNA, IGF-1, bioinformatics, osteoarthritis (OA)

## Abstract

Extracellular vesicles (EVs) have been found to have the characteristics of their parent cells. Based on the characteristics of these EVs, various studies on disease treatment using mesenchymal stem cell (MSC)-derived EVs with regenerative activity have been actively conducted. The therapeutic nature of MSC-derived EVs has been shown in several studies, but in recent years, there have been many efforts to functionalize EVs to give them more potent therapeutic effects. Strategies for functionalizing EVs include endogenous and exogenous methods. In this study, human umbilical cord MSC (UCMSC)-derived EVs were selected for optimum OA treatments with expectation via bioinformatics analysis based on antibody array. And we created a novel nanovesicle system called the IGF-si-EV, which has the properties of both cartilage regeneration and long-term retention in the lesion site, attaching positively charged insulin-like growth factor-1 (IGF-1) to the surface of the UCMSC-derived Evs carrying siRNA, which inhibits MMP13. The downregulation of inflammation-related cytokine (MMP13, NF-kB, and IL-6) and the upregulation of cartilage-regeneration-related factors (Col2, Acan) were achieved with IGF-si-EV. Moreover, the ability of IGF-si-EV to remain in the lesion site for a long time has been proven through an ex vivo system. Collectively, the final constructed IGF-si-EV can be proposed as an effective OA treatment through its successful MMP13 inhibition, chondroprotective effect, and cartilage adhesion ability. We also believe that this EV-based nanoparticle-manufacturing technology can be applied as a platform technology for various diseases.

## 1. Introduction

Osteoarthritis (OA) is a degenerative arthropathy that affects a large number of patients worldwide, resulting in poor quality of life for patients, with clinical symptoms such as pain, limited mobility, and joint deformities [1]. It is a complex chronic disease known to affect the entire joint and is characterized by inflammation, subchondral bone sclerosis, and cartilage breakdown [2]. Traditional OA treatments are aimed at alleviating pain, not complete removal of the lesion [3]. A variety of treatment approaches have been proposed for OA, including conservative treatments [4,5,6,7]. Currently, the widely performed clinically approved therapeutics include types of corticosteroids, hyaluronic acid (HA), and polynucleotides (PN) for palliative purposes. Although injection of corticosteroid provides quick relief from osteoarthritis pain, it can cause problems such as accelerated osteoarthritis progression and joint destruction [8]. Synovian^®^, a highly cross-linked HA, has been shown to lubricate joints and has good stability and effectiveness [9]. However, it has the disadvantage of requiring periodic repeat injections. Conjuran^®^, a PN injection, has been shown to have similar pain reduction efficacy to HA, with faster pain relief than HA [10,11], but PN injections also suffer from the same problem as repeated injections of HA. Currently, attempts are being made to develop promising biomaterial-based therapeutics to overcome the limitations of existing OA treatments [12].

The extracellular vesicles (EVs) are nanosized particles similar to liposomes secreted by cells and are known to reflect the characteristics of their parent cells [13,14]. In particular, EVs secreted from mesenchymal stem cells (MSCs) have regenerative activities against diseases, similar to the characteristics of MSCs [15,16,17]. In addition, EVs are non-immunogenic, allowing them to penetrate hard-to-reach areas such as the blood–brain barrier (BBB) [18]. With these properties, EVs are currently being applied to a variety of diseases, with clinical trials in progress for metastatic pancreatic cancer, COVID-19, Alzheimer’s-disease-induced dementia, and others [19]. In addition to utilizing the therapeutic properties of EVs, research on using EVs as carriers for drug delivery system is increasing [20]. Several recent studies have demonstrated the efficacy of EVs in treating OA [21,22]. In this study, we utilized EVs as the carrier to introduce siRNA, which inhibits a key OA trigger, MMP13. The MMP13 causes an imbalance between the synthesis and degradation of collagen in the joint, which leads to progressive damage to articular cartilage. Depending on MMP13-mediated successive cartilage-degrading effects on damaged cartilage, OA suffers from a vicious cycle of continual exacerbation. The small interfering RNAs (siRNA) used to downregulate MMP13 are double-stranded RNAs composed of nucleotides that take advantage of the RNA interference to repress gene expression in a sequence-specific manner [23,24]. With the possibility of clinical approval following the FDA approval of a genetic disease treatment using siRNA, an siRNA-loaded EV was used to treat the worsening of OA caused by MMP13 [25].

**Figure 1:**
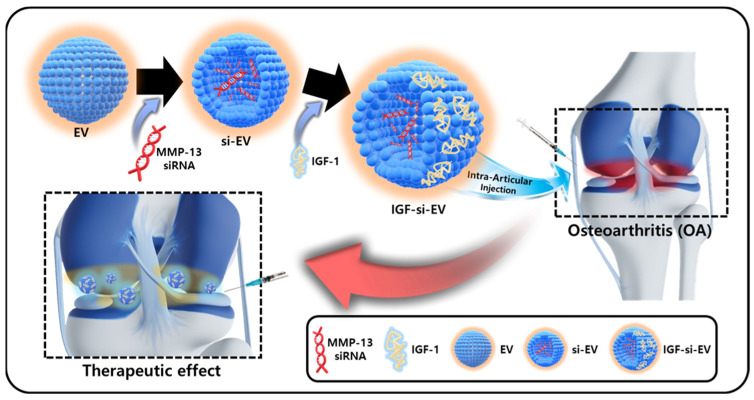
The schematic illustration of IGF-si-EV fabrication and application to osteoarthritis therapy.

Additional critical points to develop OA treatments include ensuring that the therapeutic agent remains at the lesion site for a long time so that the activity of various bioactive candidates can be sustained. To modulate the surface properties of EVs, many studies are changing the surface characteristics of EVs by attaching a ligand to the surface of EVs or through genetic manipulation at the cell level that secretes EVs [26]. However, in the case of MSCs, the complex bioconjugation process and low genetic manipulation efficiency are considered barriers to effective surface control [27]. Therefore, in this study, we attempted to engineer the surface of EVs with functional proteins without employing a complex bioconjugation strategy and gene editing techniques. Surface modification was achieved via a simple charge–charge interaction between the negatively charged EV and the positively charged insulin-like growth factor-1 (IGF-1). By utilizing the characteristics of IGF-1, which has a positive charge at pH 7.4, it can be attached to the surface of EVs through electrostatic interaction [28]. In addition to these electrostatic properties, IGF-1 is also a therapeutic factor that has been shown to enhance cell differentiation and gene expression in the extracellular matrix, while also modulating inflammation in OA [29]. We believe that the strategy of coating the surface of siRNA-loaded EVs with IGF-1 could be suitable for maintaining long-term retention in the lesion area and maximizing the regenerative effect (Figure 1).

## 2. Results and Discussion

### 2.1. Characterization of Extracellular Vesicles (EVs)

**Figure 2:**
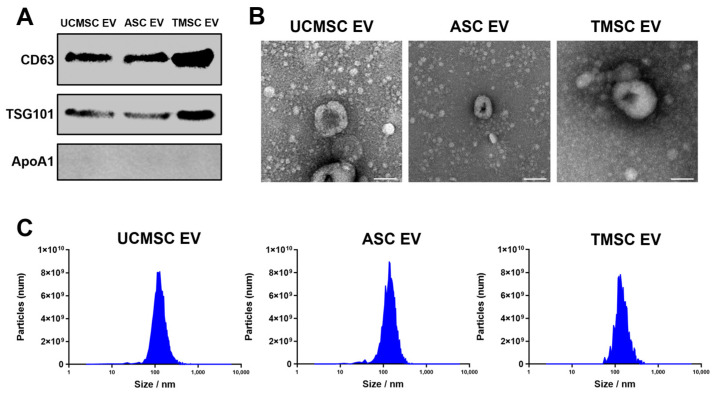
Characterization of EVs. (**A**) The Western blot analysis of an Ev derived from UCMSC, ASC, and TMSC with specific markers, CD63 and TSG101, and the negative marker ApoA1; (**B**) the morphologies of an EV imaged with TEM (scale bars equal to 100 nm); (**C**) the size distribution of an EV derived from UCMSC, ASC, and TMSC detected with Zetaview^®^ (PMX-120, Particle Metrix, Meerbusch, Germany).

The extracellular vesicles (EVs) derived from mesenchymal stem cells (MSCs) have been utilized to facilitate tissue regeneration with anti-inflammation, anti-apoptosis, and antifibrosis activities [30]. Based on the regenerative effects of EVs, a comparative analysis was conducted to select MSCs secreting EVs that exhibit optimum cartilage regeneration effects for osteoarthritis (OA) treatment. To eliminate the unknown effects from FBS-derived EVs, chemically defined media (CDM) were used to culture three different types of MSC: UCMSC, ASC, and TMSC [31]. And highly purified EVs—without FBS contaminant—were isolated from three different MSCs using a tangential flow filtration (TFF) system with a well-organized protocol for EV isolation from our lab [32,33]. When the expression of specific proteins was analyzed according to the MISEV 2023 guidelines, the representative transmembrane (CD63) and intracellular protein (TSG101) were clearly detected, whereas one of the major negative markers, apolipoprotein A1 (Apo-A1), was not expressed in either type of EV (Figure 2A). Moreover, the artefactual cup-shaped morphologies were visualized in TEM images (Figure 2B), with no significant differences in the size and number of EVs between the three types of Eve observed (Figure 2C) [34].

### 2.2. Comparative Analysis for Internal Composition of EVs

**Figure 3:**
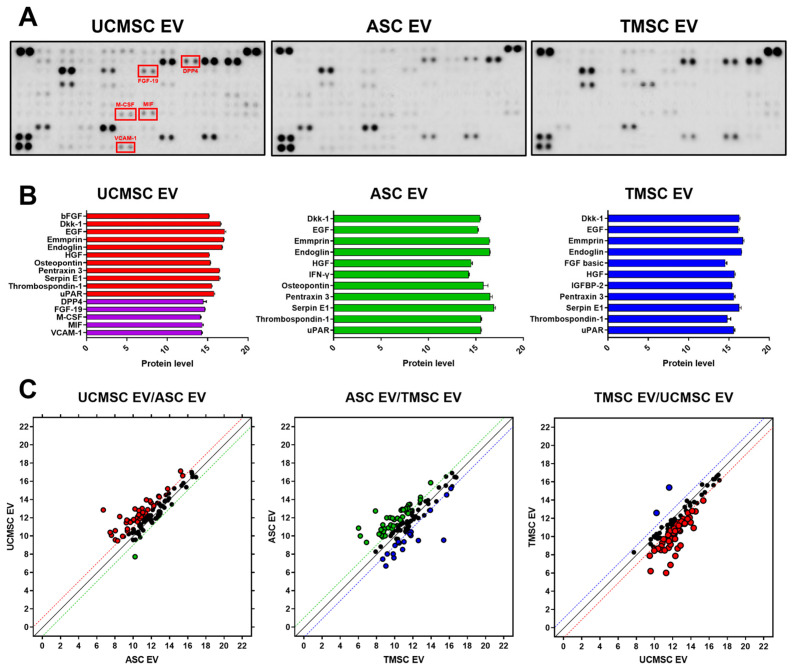
Internal characteristics of EVs: (**A**) the images for membranes of antibody array with an EV derived from UCMSC, ASC, and TMSC; (**B**) the intensities of highly expressed factors in the membranes of antibody array (intensity > 14); (**C**) comparative analysis for scatter plot of factors expressed in each EV.

The EVs are known to represent the characteristics of parent cells via paracrine effects. The internal components of EVs were analyzed using an antibody array method to verify the functionalities of EVs on the type of MSCs (Figure 3A). Figure 3B displays the factors with an intensity over 14. As shown in dickkopf-1 (Dkk-1), hepatocyte growth factor (HGF), and thrombospondin-1 (THBS1), the expression levels of most cytokines related to regeneration were similar. Dkk-1 is known to be a protein involved in Wnt signaling, and its inhibition of Wnt/β-catenin signaling may reduce the worsening effects of OA [35]. HGF, like its receptor c-Met, is widely expressed in osteoarticular tissues and may exert regenerative effects by contributing to cell motility, differentiation, and tissue morphogenesis [36]. In addition, THBS1 has therapeutic effects on OA by suppressing inflammation, although it does not directly protect chondrocytes [37]. As shown in Figure 3C, comparing the cytokines for three types of the MSC-derived EV, UCMSC-derived EVs highly expressed most regeneration-related factors, and their contents were higher than those of ASC- and TMSC-derived EVs. In the graph, the dots on the y = x line represent equally expressed factors, and the dotted lines with colors indicate factors with significant differences in expression. Therefore, the factors farther away from the y = x line and beyond the dotted line with colors showed significant differences in expression levels between the comparison groups. In detail, various regeneration-related factors, such as dipeptidyl peptidase 4 (DPP4), fibroblast growth factor 19 (FGF19), macrophage colony-stimulating factor (M-CSF), macrophage migration inhibitory factor (MIF), and vascular cell adhesion molecule 1 (VCAM-1), were upregulated in UCMSC-derived EVs. DPP4 is one of the anti-inflammatory factors in pathological conditions. Increased expression of DPP4 in rheumatoid arthritis (RA) has been shown to reduce pro-inflammatory cytokines, and it is known to exert anti-inflammatory and glycemic effects through protein–protein interactions [38]. FGF signaling is known to play an important role in maintaining cartilage homeostasis. Because dysregulation of this pathway contributes to the progression of OA, FGF-19 could be effective in maintaining cartilage homeostasis [39]. In this regard, MSC-derived EVs that can interact with FGFR1, which is overexpressed in OA patients, showing a potential therapeutic effects for OA [40,41]. Due to the properties of M-CSF to inhibit pain and cartilage damage in OA injury models [42], modulating c-FMS, the receptor for M-CSF, has been demonstrated to have therapeutic effects on OA through this pathway [43]. MIFs could play a protective role in OA by promoting the formation of the extracellular matrix (ECM), which could have potential benefits for cartilage regeneration [44]. Finally, VCAM-1 may play a role in enhancing cell migration [45]. Due to the overexpression of various factors related to cartilage regeneration, MSC-derived EVs, especially UCMSC-derived EVs, can be expected to show excellent effects in treating OA.

### 2.3. Bioinformatics Analysis for Estimating EV Functionality

**Figure 4:**
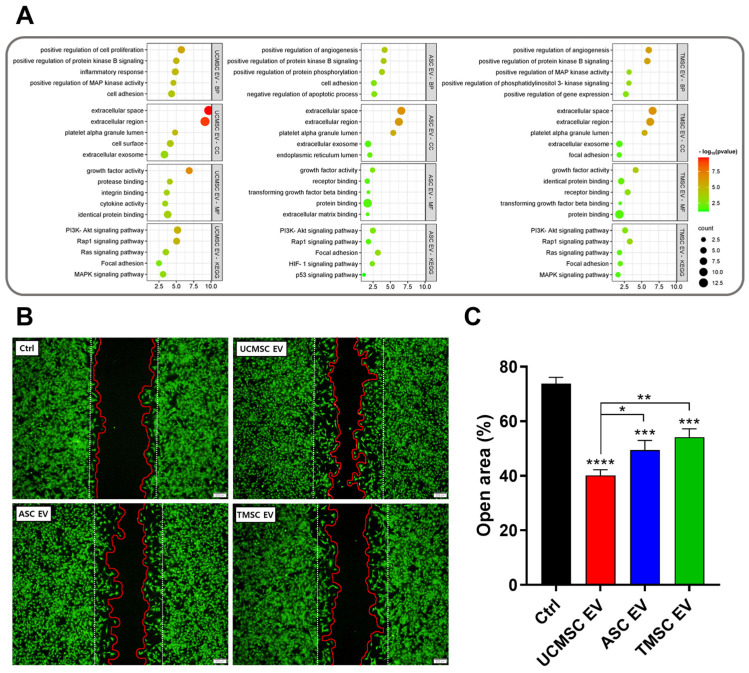
The bioinformatics analysis to select optimum EVs for osteoarthritis therapy: (**A**) the results of GO-BP, GO-CC, GO-MF, and KEGG of DAVID analysis based on high expression factors on an antibody array; (**B**) the images of the wound healing analysis with EVs derived from UCMSC, ASC, and TMSC (scale bars equal to 200 μm); (**C**) the quantified graph of wound healing analysis. (Values are presented as mean ± SD (n = 3), and statistical significance was obtained with one-way analysis of ANOVA with Tukey’s multiple comparison post-test (* *p* < 0.05; ** *p* < 0.01; *** *p* < 0.001; **** *p* < 0.0001).)

To further predict the functional aspects of the aforementioned EVs, we utilized the database for annotation, visualization, and integrated discovery (DAVID) with GO terms and KEGG pathways identification. The DAVID is a bioinformatics resource system that provides functional annotation and enrichment analysis of various factors and gives various information on small molecule–gene interactions, drug–gene interactions, tissue expression information, disease information, and related pathways [46]. Based on the highly expressed proteins of three types of EVs (UCMSC EV, ASC EV, and TMSC EV) selected by the antibody array, we utilized the DAVID to identify biological processes (BP), cellular components (CC), and molecular functions (MF), as well as the Kyoto Encyclopedia of Genes and Genomes (KEGG) (Figure 4A). The GO-CC of all EVs commonly includes elements such as “extracellular space”, “extracellular area”, and “extracellular exosome”, indicating that the EVs are actually extracellular components released from cells. In GO-MF terms, factors such as “growth factor activity”, “receptor binding”, and “cytokine activity” are identified. The “growth factor activity” is involved in the delivery of biologically active molecules to cells and tissues, which increases the therapeutic efficacy with regenerative effects of EVs [47]. The “receptor binding” can refer to its role in delivering to specific cells and tissues [48]. The cytokines in “cytokine activity” are important regulators of immune response and inflammation [49]. The cytokines can bind to receptors on cells and regulate functions such as secretion, proliferation, apoptosis, and the differentiation of cells [50]. Noteworthy in the GO-MFs, “growth factor activity” for UCMSC-derived EVs had the highest *p*-value across all GO-MFs. Based on these results, UCMSC-derived EVs may be more advantages for delivery to cells and tissues. The proposed BPs included “positive regulation of cell proliferation”, “positive regulation of protein kinase B signaling”, “inflammatory response”, “negative regulation of apoptotic process”, and “cell adhesion”. The “positive regulation of cell proliferation” can be expected to have a therapeutic effect through the activity of cell proliferation [51]. Protein kinase B signaling plays a crucial role in a variety of biological processes, and protein kinase B is also known as AKT. The AKT is a factor that promotes chondrocyte proliferation and autophagy, which is essential for the maintenance and repair of cartilage [52]. There are numerous studies that indicate that inflammation-inducing factors are ameliorated by EVs, which could contribute to the inflammatory response [53,54]. Apoptosis is a type of “programmed cell death” a natural phenomenon, but it can be triggered by factors such as ROS [55]. This apoptosis can contribute to inflammation under certain conditions; on the other hand, NF-kB, a hallmark of inflammation, can trigger apoptosis by regulating proapoptotic factors [56]. Various studies have shown that EVs can regulate NF-kB and thus inhibit apoptosis [57]. The “cell adhesion” is a fundamental component of biological processes such as cell growth, differentiation, migration, and tissue formation, and its importance may play a role in inhibiting disease progression and treatment [58]. The KEGG results showed the “PI3K-Akt signaling pathway”, “Rap1 signaling pathway”, “Ras signaling pathway”, etc. The “PI3K-AKT signaling pathway” is known to regulate a variety of biological activities, including cell division, autophagy, survival, and differentiation [59]. The “Rap1 signaling pathway” can contribute to cell adhesion, and the “Ras signaling pathway” can affect cell proliferation and differentiation [60,61]. Taken together, the results of GO-BP, GO-CC, GO-MF, and KEGG of UCMSC-, ASC-, and TMSC-derived EVs allowed us to hypothesize that EVs may have a therapeutic effect on OA.

### 2.4. Evaluation of Wound-Healing Effects of EVs

A wound healing assay was performed using primary rat chondrocytes (PRCs) to verify the regenerative ability of EVs on OA predicted by bioinformatics analysis (Figure 4B). Wound closure rates of scratch-formed PRCs were monitored 24 h after incubating equal amount of EVs. When the morphology of calcein AM-stained viable cells was analyzed, the open area was approximately 74% in the Ctrl group, 40% in the UCMSC EV group, 49% in the ASC EV group, and 54% in the TMSC EV group, respectively (Figure 4C). The results of these wound healing assays exhibited that the strong wound-healing ability of UCMSC-derived EVs are correlated with the results of antibody-array-based bioinformatics analysis.

### 2.5. Confirming Optimum siRNA for MMP13 Inhibition

**Figure 5:**
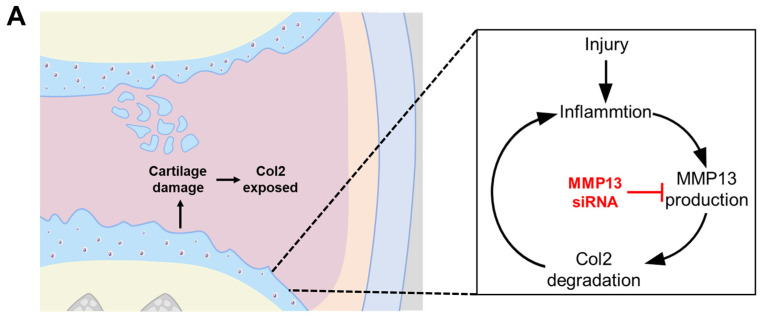

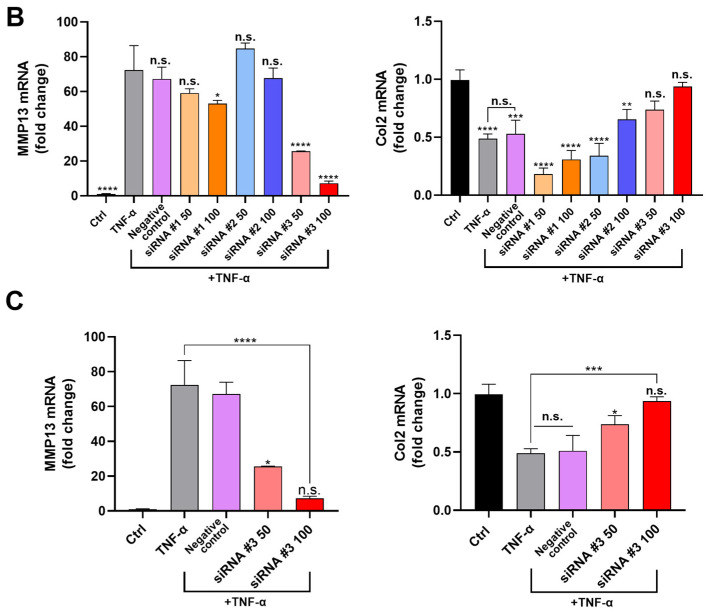
Selecting the optimum MMP13 siRNA: (**A**) the illustration of OA progress by MMP13-induced inflammation and Col2 degradation cycle and the role of siRNAs to inhibit the progression of OA; (**B**) evaluation of the inhibitory activities of siRNA #1, siRNA #2, and siRNA #3 against MMP13; (**C**) concentration-dependent inhibitory efficacy of siRNA #3 against MMP13. (Values are presented as mean ± SD (n = 3), and statistical significance was obtained with one-way analysis of ANOVA with Tukey’s multiple comparison post-test (n.s. > 0.05; * *p* < 0.05; ** *p* < 0.01; *** *p* < 0.001; **** *p* < 0.0001).)

The MMP13 is a critical targeting gene causing the progression of OA [62]. The degenerative cycle of OA causes cartilage to become damaged and inflamed, leading to the increased expression of matrix metalloproteinase-13 (MMP13) and the subsequent degradation of collagen type 2 (Col2). This vicious cycle is perpetuated by MMP13 and can be downregulated by siRNA of MMP13 (Figure 5A). The siRNAs are double-stranded RNAs that utilize RNA interference to target disease-related genes [63]. The siRNA-based therapies are currently approved by the FDA, and the therapeutic products have been released as Patisiran, Givosiran, Lumasiran, Inclisiran, and Vutisiran [64]. Based on these, siRNAs were selected to inhibit MMP13 for efficient suppression of OA. To confirm the MMP13 inhibitory effects for three different sequences of siRNAs on TNF-α (20 ng/mL)-pretreated PRCs, the RNAs from PRCs were extracted 24 h after siRNA treatment and the gene expression levels of MMP13 and Col2 were analyzed using the qPCR method [3,65]. The results showed that siRNA #3 had the highest MMP13 inhibition effect with the protective effect for Col2 (Figure 5B). The siRNA #3 displayed a concentration-dependent inhibitory effect, and the maximized MMP13 inhibition efficiency was reached at approximately 90% in the group of 100 nM siRNA #3 compared to TNF-α-treated group (Figure 5C). With these, siRNA #3 was selected as the optimum MMP13 siRNA to show the highest MMP13 inhibition and Col2 protection effects.

### 2.6. Fabrication and Characterization of si-EV and IGF-si-EV

**Figure 6:**
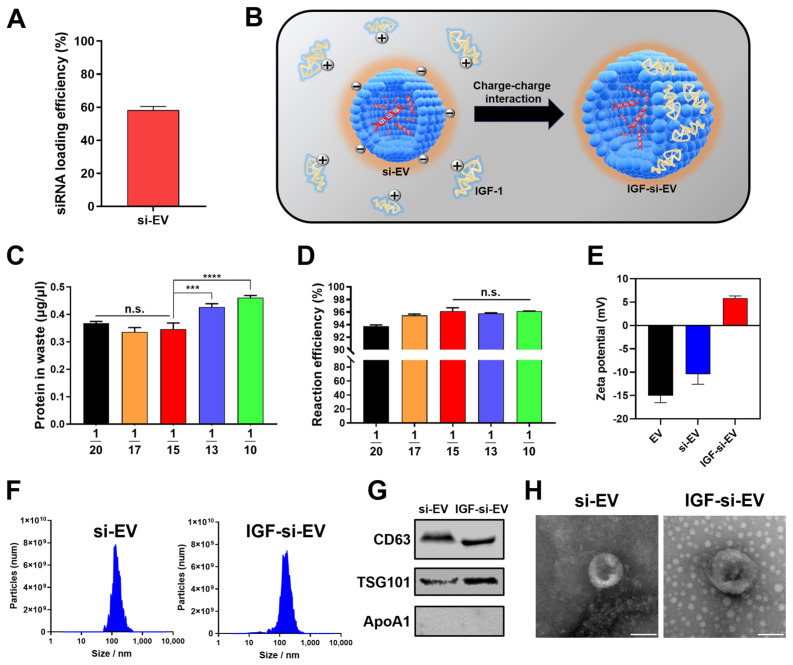
Functionalization and characterization of EVs: (**A**) the loading efficiency of siRNA into EVs; (**B**) the schematic image of fabrication for IGF-si-EVs; (**C**) the concentration of waste IGF-1 depending on the ratio of si-EV and IGF-1; (**D**) the calculated reaction efficiency of IGF-si-EVs depending on the ration of si-EV and IGF-1; (**E**) the zetapotential of EV, si-EV, and IGF-si-EV; (**F**) the size distribution of si-EVs and IGF-si-EVs measured with Zetaview^®^; (**G**) the Western blot analysis to confirm si-EV and IGF-si-EV (positive markers of EV: CD63, TSG101; negative marker of EV: ApoA1); (**H**) the morphologies of si-EV and IGF-si-EV imaged with TEM (scale bars equal to 100 nm). (Values are presented as mean ± SD (n = 3), and statistical significance was obtained with one-way analysis of ANOVA with Tukey’s multiple comparison post-test (n.s. > 0.05; *** *p* < 0.001; **** *p* < 0.0001).)

To load siRNA #3 into EVs, Exo-Fect™ Exosome Transfection Kit (Exo-Fect) was utilized. The Exo-Fect is a commercially available method for loading small RNAs inside EVs efficiently and delivering them to target cells [66]. The siRNA #3 loading efficiency reached 58% (Figure 6A), and IGF-1 was intended to be attached onto the surface of si-EVs to make IGF-si-EVs a final candidate for OA. The IGF-1 is a vital factor in cartilage regeneration and has been shown to have therapeutic effects on OA by facilitating chondrogenic differentiation and proliferation of MSCs, as well as protective effects against OA by regulating various signaling processes to promote cartilage repair and regeneration [67]. Moreover, the IGF-1 is known to have an isoelectric point (pI) of 8.5, which displays a cationic property at a neutral pH (7.0) [28], allowing it to attach to the surface of negatively charged EVs and to be captured by cartilage tissues composed of negatively charged chondroitin sulfate simultaneously [68] (Figure 6B). To optimize the ratio of si-EV and IGF-1 for IGF-si-EVs fabrications, five different concentrations of IGF-1 were incubated with a fixed concentration of si-EV (at ratios of 1/20, 1/17, 1/15, 1/13, and 1/10 of the protein concentration in si-EV). Continuously, IGF-si-EV was washed using an Amicon Ultra-15 centrifugal filter unit, and the concentration of unreacted IGF-1 was measured to select the optimum ratio of si-EV and IGF-1 for IGF-si-EVs (Figure 6C). And the reaction efficiency was also calculated based on the amount of initially added IGF-1 and measured waste (Figure 6D). With the amount of waste and reaction efficiency, the optimum reaction amount of IGF-1 was determined to be 1/15 for the protein concentration of EV. Finally, the surface charge of IGF-si-EV was found to be cationic due to the cationic nature of IGF-1, which is supported by the fact that it shows a similar tendency to the surface charge of conventional surface-modified PLGA nanoparticles with IGF-1 [69] (Figure 6E). The average size slightly increased with the addition of IGF-1 (166 nm at si-EV and 191 nm at IGF-si-EVs, respectively) (Figure 6F). To confirm that si-EV and IGF-si-EV originated from UCMSC-derived EVs and maintained the characteristics of those EVs, the expression of representative proteins and morphologies was serially evaluated using Western blot and TEM, respectively (Figure 6G,H).

### 2.7. Regenerative Activities of Nanovesicle

**Figure 7:**
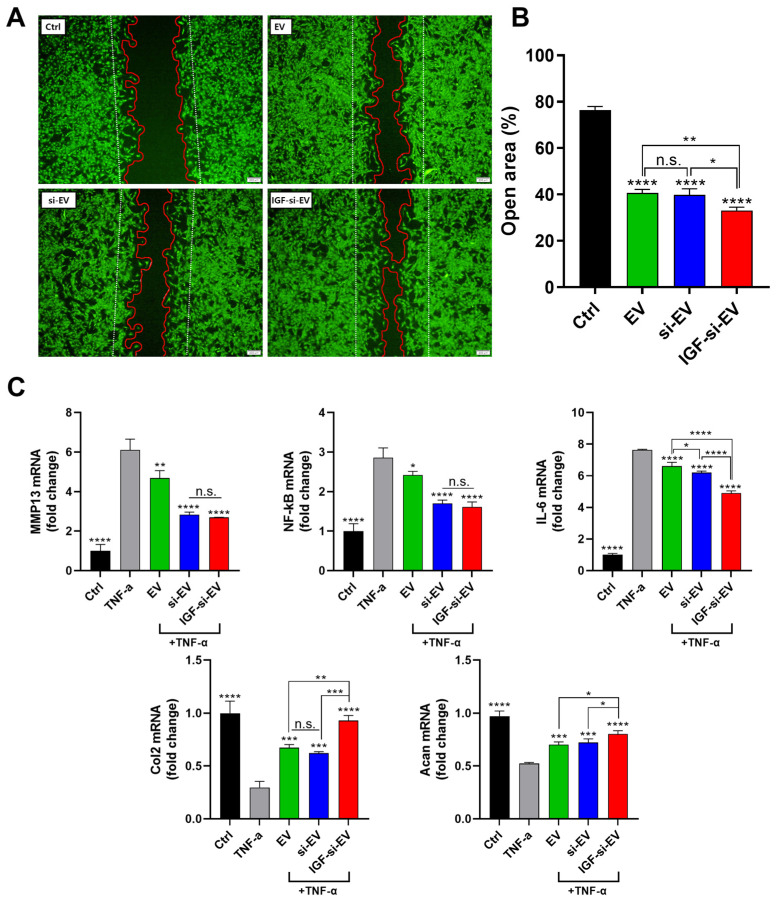
Therapeutic effects of EVs, si-EVs, and IGF-si-EVs on damaged chondrocyte in vitro: (**A**) the wound-healing effects of EVs, si-EVs, and IGF-si-EVs; (**B**) the quantification graph of the wound healing assay; (**C**) the gene expression levels of inflammatory cytokines and regeneration factors to confirm the therapeutic effect on OA with EVs, si-EVs, and IGF-si-EVs. (Values are presented as mean ± SD (n = 3), and statistical significance was obtained with one-way analysis of ANOVA with Tukey’s multiple comparison post-test (n.s. > 0.05; * *p* < 0.05; ** *p* < 0.01; *** *p* < 0.001; **** *p* < 0.0001).)

To confirm the regenerative properties for all types of nanovesicles (EV, si-EV, and IGF-si-EV) on cartilage, we performed a wound healing assay using PRCs (Figure 7A). This assay was performed to determine the basal regenerative function of the particles. Twenty-four hours after particle treatment, EVs and si-EVs showed similar wound-healing effects with about 40% open area (Figure 7B). This suggests that MMP13 siRNA does not appear to have an additional effect on cartilage regeneration. But the cartilage promotion ability of IGF-1 can be proven through the enhanced wound-healing effects of IGF-si-EVs compared to EVs and si-EVs. And these are due to the cartilage-regeneration-related gene expression of IGF-1 [70]. The inflammation inhibitory effects of nanovesicles were additionally demonstrated in TNF-α induced RPCs to mimic the condition of OA. In the TNF-α-pretreated OA condition for the in vitro model, the expression of MMP13 was significantly downregulated in the si-EV and IGF-si-EV groups (Figure 7C). However, the inhibition of MMP13 was not different between these two groups (si-EV and IGF-si-EV), suggesting that the suppression of MMP13 is due to siRNA rather than IGF-1. The inhibitory effects for the other proinflammatory cytokines, NF-kb and IL6, demonstrated similar trends to MMP13. These results are supported by a previous report that IGF-1 has the effect of alleviating inflammatory factors [71]. Contrary to these, the expression levels of the representative cartilage components, Col2 and Aggrecan (Acan), showed moderate improvements in both EVs and si-EVs, and were significantly enhanced in IGF-si-EVs, which is believed to be a secondary effect of the MMP13-inhibitory effects for IGF-1. Furthermore, IGF-si-EVs show significant differences from EVs and si-EVs in the expression of Col2 and Acan. The results of the wound healing assay in the OA-mimicked PRC test suggested that IGF-si-EVs could be an optimum treatment for OA.

### 2.8. Affinity to Femoral Condyle of IGF-si-EV

**Figure 8:**
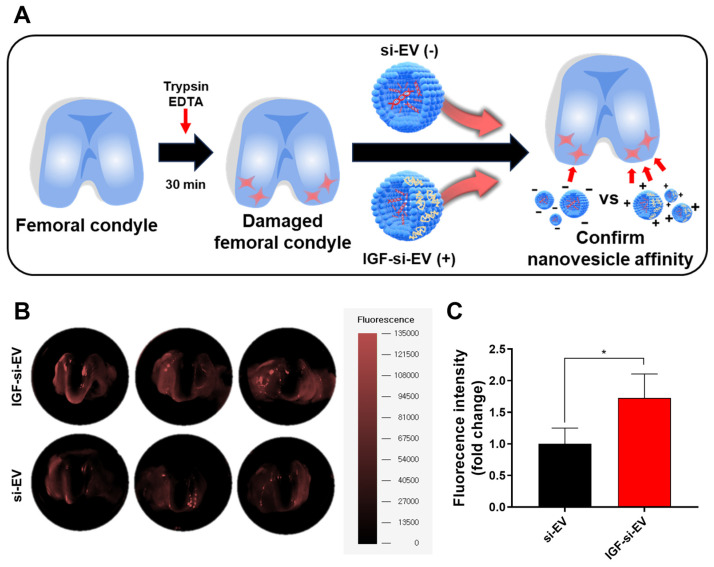
The ex vivo study to compare nanovesicle affinity: (**A**) the illustration of ex vivo model system to mimic the OA model and the nanovesicle affinity test with extraction of damaged knee cartilage; (**B**) the image of nanovesicle affinity with damaged femoral condyle imaged with fluorescence-labeled organism-bioimaging instrument (FOBI); (**C**) the graph of the analyzed fluorescence intensities. (Values are presented as mean ± SD (n = 3), and statistical significance was obtained with one-way analysis of ANOVA with Tukey’s multiple comparison post-test (* *p* < 0.05).)

With therapeutic effects to injured cartilage, long-term retention at the lesion site is also very important in developing therapeutics for OA because the vesicles can be rapidly cleared by synovial vasculature or lymphatic drainage with intra-articular treatment [72]. To overcome this, typical intra-articular treatments were developed with micro size, and hydrogels were utilized for sustained release, or charge–charge interactions with cartilage were leveraged. Among these strategies, a method for increasing affinity via charge–charge interaction between IGF-1 and the lesion site was selected, and to prove this, the affinity between nanovesicles and damaged femoral condyle was comparatively analyzed. The femoral condyles isolated from rats were incubated in Trypsin EDTA for 30 min to construct an ex vivo OA model. The equal amounts of DiD-labeled si-EVs and IGF-si-EVs (1 × 10^10^ particles) were treated in the constructed ex vivo model, and the fluorescent intensity was verified at 30 min later using FOBI (Figure 8A). The intensity of the red fluorescent signal was stronger in femoral condyles treated with IGF-si-EVs, and a clear difference can be verified in the graph quantifying the fluorescence intensity (Figure 8B,C). With this, the therapeutic effects of IGF-si-EVs are expected to last for a long time due to the interaction with damaged chondrocyte via the characteristic of positive charging by IGF-1.

## 3. Materials and Methods

### 3.1. Cell Culture

Human-umbilical-cord-derived mesenchymal stem cells (UCMSCs; CHA Biotech Co., Ltd., Seongnam, Republic of Korea), human-adipose-derived mesenchymal stem cells (ASCs; Lonza, Basel, Switzerland), and human-tonsil-derived mesenchymal stem cells (TMSCs; presented from the Dong-Youn Hwang’s Laboratory in CHA University) were cultured using CellCor^TM^ CD MSC media (CDM; Xcell Therapeutics, Seoul, Republic of Korea) with 1% antibiotic–antimycotic solution. All types of MSCs were seeded at 5 × 10^5^ cells/plate on a 150 pi plate. The primary rat chondrocyte (PRC; presented from Dr. Byoung Ju Kim) was cultured using DMEM-F12 (Gibco, Grand Island, NY, USA) media with 1% antibiotic–antimycotic solution and 10% fetal bovine serum (FBS). All cell types were incubated at 37 °C in a humidified environment with 5% CO_2_.

### 3.2. Cell Viability Assay

The cell viability was determined using the Cell Counting Kit-8 (CCK-8; Dojindo, Kumamoto, Japan). The CCK-8 testing was carried out in accordance with the manufacturer’s instructions to determine relative cell viability. At 450 nm, absorbance was measured using a microplate reader (Molecular Devices, Silicon Valley, CA, USA).

### 3.3. Extracellular Vesicles (EVs) Isolation

The conditioned media with CDM were collected every 24 h for a total of 120 h in order to isolate EV. The collected CDM were centrifuged at 1300 rpm for 3 min, followed by elimination of the non-exosomal large particles, such as cells, cell debris, microvesicles, and apoptotic bodies, using a 0.22 μm vacuum filter/storage bottle system. A 500 kDa-molecular-weight cut-off filter was used in tangential flow filtration (TFF; Repligen, Waltham, MA, USA) to isolate EVs. The rate of diafiltration was adjusted at 7. The Amicon Ultra-15 centrifugal Filter Unit (Merck, Darmstadt, Germany) was used to concentrate the isolated EVs for further applications.

### 3.4. Characterization of EV and Particles

The MONO ZetaView^®^ (PMX-120, Particle Metrix, Meerbusch, Germany) was used with a 488 nm scatter mode to validate the quantity and size of particles. Using filtered phosphate-buffered saline (PBS) solution (HyClone Laboratories, Logan, UT, USA), the EV samples were diluted to 10^7^–10^8^ particles/mL. For all samples, the detailed parameters for accurate analysis were tuned with sensitivity 75, shutter 100, minimum trace length 15, and cell temperature 25 °C. The zeta potential of particles was identified with Zetasizer Nano ZS (Malvern, Worcestershire, UK). To visualize the morphologies of EVs, transmission electron microscopy (TEM; Hitachi, H-7600, 80 kV, Tokyo, Japan) was used. The EV solution was dried on a Formvar/copper grid with a carbon coating of 150 mesh (FCF150-CU, Electron Microscopy Sciences, Hatfield, PA, USA). EVs were stained with 7% uranyl acetate or gadolinium acetate solution and dried for negative staining on a copper grid. The Formvar/copper grid was put on the grid box for TEM investigation after drying.

### 3.5. Western Blot Analysis

In Western blot analysis, the same number of EVs was used for parallel comparison og protein expression levels. The EVs were placed onto nitrocellulose (NC) membranes after separation with 10% SDS-PAGE. The NC membrane was blocked using a TBST solution diluted in 5% skim milk. The EV protein-transferred NC membranes were incubated with CD63 (Abcam, Waltham, MA, USA), TSG101, and Apo-A1 primary antibodies (Santa Cruz Biotechnology, Santa Cruz, CA, USA), followed by HRP-linked secondary antibodies (Cell Signaling Technology, Danvers, MA, USA). The blot was pretreated with the enhanced chemiluminescence solution (GE Healthcare, Milwaukee, WI, USA) and visualized with ChemiDoc^TM^ XRS+ and ImageLab software 6.0.1 (Bio-Rad, CA, USA).

### 3.6. Antibody Array

Radioimmunoprecipitation assay (RIPA; Rockland Immunochemicals, Pottstown, PA, USA) buffer was used to lyse the same number of EVs. The lysed EV solutions were placed onto the NC membrane of the Proteome Profiler^TM^ Antibody Arrays Human XL Cytokine Array Kit (R&D Systems, Minneapolis, MN, USA). After developing the NC membrane with ChemiDoc^TM^ XRS+, the intensity of the antibody array was assessed using ImageLab software 6.0.1. For data analysis, the average intensity of expression was employed. The data were expressed as antibody array intensities in logarithm base 2. The GO terms and KEGG pathway terms were analyzed using the DAVID (database for annotation, visualization, and integrated discovery; https://david.ncifcrf.gov/; accessed on 1 February 2024). The graphs of GO terms and the KEGG pathway were obtained with SRplot (https://www.bioinformatics.com.cn/srplot; accessed on 4 April 2024).

### 3.7. Scratch Wound Healing Assay

With 5 × 10^5^ cells per well, 6-well plates were used for the PRC culture. The cells were cultivated until confluent with a monolayer. A sterilized 1 mL pipette tip was then used to scratch the cells on the center of the wells. The cells were treated with the same concentration of nanovesicles (1 × 10^8^ particles/mL) after being rinsed with PBS solutions while labeling cells with calcein AM solution. The calcein-AM-labeled cells were visualized with a fluorescence microscope after 24 h to monitor wound-healing activities. The ImageJ 1.47t (Wayne Rasband, NIH, Bethesda, MD, USA) plugin for the wound healing tool was utilized to calculate the percentage of open area.

### 3.8. Selecting the Optimum MMP13 siRNA

Three types of candidates for siRNA sequences were first screened in PRCs stimulated with the inflammatory cytokine TNF-α (20 ng/mL) to target different sites of the MMP13. Three different antisense sequences, namely, siRNA #1—UUG GUU UUC UCA UGA UGU C, siRNA #2—ACA UGG UUG GGA AGU UCU G, siRNA #3—UAC UGA AGC UUGUUC ACA G, were used. The siRNA for negative control was utilized by AccuTarget™ negative control siRNA (NC siRNA). Oligonucleotides used in these studies were purchased from Bioneer (Bioneer, Daejeon, Republic of Korea). The 50 nM, 100 nM siRNAs, and 100 nM NC siRNA were applied to TNF-α-pretreated PRCs. For an efficient delivery of oligonucleotide to primary cells, the Lipofectamine RNAiMAX transfection reagent (Thermo Fisher Scientific, Waltham, MA, USA) was used. The RNA of the PRCs was extracted after 24 h to evaluate the expression level of the MMP13. The optimum sequence of siRNA was selected by comparing expression levels of MMP13.

### 3.9. Synthesis of siRNA Loaded EVs (si-EVs) and IGF-si-EVs

To load siRNA into EVs, the Exo-Fect™ Exosome Transfection Kit (System Biosciences, Palo Alto, CA, USA) was utilized. The loading procedure of siRNA into the EV was carried out in accordance with the manufacturer’s instructions. The siRNA loading efficiency was assessed by evaluating the concentration of siRNA in waste detected with NanoDrop™ One (Thermo Fisher Scientific, Waltham, MA, USA). The protein concentration of the fabricated si-EV was determined with the Pierce™ BCA Protein Assay Kit (Pierce, Waltham, MA, USA). To modify the surface of si-EV, the proper amount of IGF-1 was added to the si-EV solution. After 1 h, the reacted solution was washed with Amicon Ultra-15 centrifugal filters (Millipore, Billerica, MA, USA) to remove unreacted IGF-1. The fabricated si-EVs and IGF-si-EVs were stored at 4 °C until use.

### 3.10. Confirming Modification of Osteoarthritis Related Factors

The PRCs were seeded at the density of 5 × 10^5^ cells/well on 6-well plates. The TNF-α (20 ng/mL) and the same number of particles were treated for 24 h simultaneously. After 24 h, RNA for qPCR (quantitative real-time PCR) was extracted to determine the level of inflammation and regeneration. The SYBR green PCR reagent mix (Applied Biosystems, Foster City, CA, USA) was applied to real-time PCR. QuantStudio 3 (Applied Biosystems, Foster City, CA, USA) was employed to perform reactions with the following primers. MMP13: forward, 5′-ctgcggttcactttgaggac-3′ and reverse, 5′-acagcatctactttgtcgcc-3′; Col2: forward, 5′-caccgctaacgtccagatgac-3′ and reverse, 5′-ggaaggcgtgaggtcttctgt-3′; Acan: forward, 5′-cattcgcacgggagcagcca-3′ and reverse, 5′-tggggtccgtgggctcacaa-3′; NF-kB: forward, 5′-ccgggatggcttctatgag-3′, and reverse, 5′-ccgtctttctgtcacggtct-3′; IL-6: forward, 5′-cctggagtttgtgaagaacaact-3′ and reverse, 5′-ggaagttggggtaggaagga-3′; 18 s rRNA: forward, 5′- gtggttttcggaactgaggc-3′ and reverse, 5′-gtcggcatcgtttatggtcg-3′. The data were quantified using 2^−ΔΔCt^ method with 18 s rRNA as a reference.

### 3.11. Ex Vivo Nanovesicle Affinity Study

The femoral condyle was extracted from the 12-weeks-old Sprague-Dawley (SD) rats. The bony part of the femur of the left leg was removed to isolate the femoral condyle only. Isolated femoral condyle was washed using PBS solution. The washed femoral condyle was exposed to 0.25% of trypsin-EDTA (TE) in 30 min. The damaged femoral condyle was washed with PBS solution after TE incubation. Before si-EV and IGF-si-EV fabrication, the natural EV was incubated with Vybrant™ DiD Cell-Labeling Solution (Thermo Fisher Scientific, Waltham, MA, USA) in 30 min to visualize EV and the DiD-labeled EVs were utilized to fabricate si-EV and IGF-si-EV. The ex vivo model manufactured to mimic osteoarthritis was exposed to the same numbers of si-EVs and IGF-si-EVs (1 × 10^10^ particles) to compare nanovesicle affinity to damaged chondrocyte. The nanovesicle affinity with femoral condyle was examined with DiD intensity using a fluorescence-labeled organism bioimaging instrument (FOBI; Neoscience, Seoul, Republic of Korea). The detected DiD intensity was normalized with the area of the femoral condyle.

### 3.12. Statistical Analysis

GraphPad Prism 7 was used to assess every statistical analysis (GraphPad Prism 7.00 Software, La Jolla, CA, USA). Unpaired *t* tests or one-way analysis of variance (ANOVA) with Tukey’s multiple comparison post-test were applied to assess differences for more than three groups. *p*-values below 0.05 were determined to be statistically significant (* *p* < 0.05; ** *p* < 0.01; *** *p* < 0.001; **** *p* < 0.0001).

## 4. Conclusions

A new type of EV-based nanovesicle, IGF-si-EV, was proposed for OA treatment. The IGF-si-EVs contain siRNA and IGF-1 that downregulate MMP13 expression and support the production of cartilage building block, respectively, based on UCMSC-derived EVs. Additionally, IGF-1-mediated cationic surface charge of IGF-si-EVs can help sustain the therapeutic effect through interaction with the negative charge of damaged chondrocytes under the conditions of a highly circulated joint cavity. Based on these results, IGF-si-EVs could be an effective treatment for OA and show promise as a platform system that can be applied to other diseases using a similar fabrication method. In addition, IGF-1 is being utilized as an FDA-approved drug for purposes other than the treatment of OA, and siRNA has recently been introduced as a therapeutic for diseases other than OA. We can expect to develop treatments for OA in the near future by introducing IGF-1 and siRNA that can be applied clinically.

## Data Availability

Data are contained within the article.
